# Improved Thermal Insulating Properties of Renewable Polyol Based Polyurethane Foams Reinforced with Chicken Feathers

**DOI:** 10.3390/polym11122002

**Published:** 2019-12-03

**Authors:** Ibon Aranberri, Sarah Montes, Ewa Wesołowska, Alaitz Rekondo, Krystyna Wrześniewska-Tosik, Hans-Jürgen Grande

**Affiliations:** 1 CIDETEC, Basque Research and Technology Alliance (BRTA), Po. Miramón 196, 20014 Donostia-San Sebastián, Spain; smontes@cidetec.es (S.M.); arekondo@cidetec.es (A.R.); hgrande@cidetec.es (H.-J.G.); 2 Institute of Biopolymers and Chemical Fibres, ul Skłodowskiej-Curie 19/27, 90-570 Łódź, Poland; e.wesolowska@ibwch.lodz.pl (E.W.); k.wrzesniewska.tosik@ibwch.lodz.pl (K.W.-T.)

**Keywords:** polyurethane biofoams, chicken feathers, thermal conductivity

## Abstract

In the present work, sustainable rigid polyurethane foams (RPUF) reinforced with chicken feathers (CF) were prepared and characterized. The bio-based polyol used to formulate the foams was obtained from castor oil. This investigation reports the influence of the chicken feathers fibers as reinforcement of RPUF, on water absorption, thermal, mechanical and morphological properties (field-emission scanning electron microscope—FESEM) and thermal conductivity on water-blown biofoams. It was found that the biofoams improved thermal insulating properties when CF was added. The addition of CF to foams provided lower heat flux density to the biofoams obtaining bio-based materials with better insulation properties. The results obtained in this study proved that the incorporation of CF to RPUF modified the cell structure of the foams affecting their physical and mechanical properties, as well as functional properties such as the heat transmission factor. These biofoams containing up to 45% of bio-based materials have shown the potential to replace fully petroleum-based foams in thermal insulation applications.

## 1. Introduction

Polyurethanes (PU) are a special group of polymeric materials that are in many ways different from most of the other plastic types. They can be incorporated into many different items, such as paints, liquid coatings, elastomers, insulators, elastic fibers, foams, integral skins, etc. [1,2]. Presently, rigid polyurethane foams represent one of the most important commercial products of PU due to their versatility and energy saving insulation properties. PU foams have advantages of lightweight, low cost and especially good adherence toward the surface of sheathing layer. More importantly, they are popular materials with excellent insulating properties for their lowest thermal conductivity over other cellular materials [3]. Hence, polyurethane foams (PUF) take up between the 20% and 25% of PU production [4]. Normally, open cells foams are appropriate for sound insulation applications, while closed cells foams are appropriate for thermal insulation purposes. The thermal insulation is primarily due to a combination of cell size and cell morphology, which trap the low thermal conductive gas inside. In addition, their high mechanical strength and their easy processing make rigid polyurethane foams (RPUF) an attractive choice in various industrial applications, such as refrigerators and sandwich panels, where RPUF dominate the market [5].

RPUF are typically synthesized by a polyol and an isocyanate, which compose the backbone urethane group. In addition, blowing agents, surfactants, catalysts and fillers are added to the formulation in order to improve the physical and mechanical properties of the RPUF. These foams can significantly reduce energy costs on one hand and can make commercial and residential appliances more comfortable and efficient on the other. The preparation of RPUF can be performed using petroleum-based polyols as well as bio-based polyols from vegetable oils. Currently, most of the polyols used in the manufacture of polyurethanes are derived from the petroleum industry. However, during the last years a huge effort is done in order to replace petroleum-based polyols by vegetable based oils such as soybean oil [6], palm oil [7] and castor oil [8]. In this way, the use of castor oil as polyol source has been proved outstanding because it is a biocompatible and hydrophobic monomer, which is composed of 90% ricinoleic fatty acid-the unique natural fatty acid with hydroxyl groups in its chain [9]. Castor oil is extracted from the seed of the castor oil plant, is not edible, which unlike soybean oil will not compete with food and can formulate biodegradable and eco-friendly polyurethanes under certain conditions [10].

Natural fibers, which are available from renewable resources, are an attractive alternative to be used as reinforcing filler in RPUF and so, increase the content of biodegradable components of biofoams. The group of A. Prociak has reported studies on rapeseed oil based RPUF with the addition of flax fibers [11] and walnut shells and microcellulose [12]. Marcovich et al. studied castor oil-based PU filled with wood flour [13] and hemp and wood fibers [14]. S. Czlonka confirmed that the addition of a certain level of potato protein might decrease the physico-mechanical properties of modified foams [15]. Y.C. Chen used bamboo charcoal in castor-oil based PU as an alternative to replace indoor building materials [16] and Zieleniewska studied RPUF reinforced with filler coming from animal waste such as 25% of egg shells [17].

The poultry industry generates a vast amount of feather waste every year, most of which ends up in landfill or goes through an energy-intensive process to be converted into low-grade animal feed. According to the European Commission 11.8 million tonnes of poultry meat were produced in the European Union (EU) in 2018 and it will gradually increase to 12.2 million tonnes by 2028 [18]. Feathers are an abundant waste of poultry industry since they account for approximately 8% of the adult chicken weight and are constituted by about 90% protein. Unfortunately, the current solutions do not exploit the opportunity that this keratinous material represents, and more importantly, the management of environmental and health concerns as overall waste rises. Incineration is not a proper solution since disposal of feathers by waste burning emit higher levels of microparticles, CO_2_, NO_2_ and CO than new coal plants [19]. Moreover, landfilling must be prevented because of its unfriendly consequences for the nearby environment, especially the contamination of surface and ground water, soil and air.

Feathers, a waste generated by the poultry industry, can be also a suitable reinforcement for RPUF Feather barbs show hollow honeycomb structures, resulting in unique properties such as low density, high compressibility, sound absorption and heat insulators [20]. Moreover, J. Bessa et al. showed diameters of the many hollow internal channels within the feather microstructure ranging from 3.12 to 10.26 µm [21]. Therefore, chicken feathers (CF) were used to improve compressive strength of bio-based RPUF reinforced with 1.5 wt % of CF [22], to obtain lightweight polymeric materials with high CF content [23,24] and thermal insulators in several applications such as mats [25] and hybrid boards [26]. The thermal conductivity of feathers is 0.034 W/(m·K) [27], slightly lower than polyurethane foams obtained from castor oil-based polyol, 0.039 W/(m·K) [13]. Therefore, a reduction of the thermal conductivity in the feather-reinforced polyurethanes could be foreseen.

To the best of our knowledge there are no studies concerning polyurethane foams with high bio-based content, combining a commercially available castor oil based polyol and reinforced with high CF content with improved thermal insulating properties. Cell morphology, thermal conductivity, mechanical properties, water absorption and thermal stability of the biofoams were investigated. 

## 2. Materials and Methods

### 2.1. Materials

Chicken feather fibers were supplied by Grupo SADA (Madrid, Spain). The feathers were first cleaned by washing with industrial alkali detergent and then dried at 60 °C for 24 h. After the drying process, chicken feathers were sterilized in an autoclave with pressurized steam treatment at 126 °C for 30 min and 30 min of drying. Microbiological tests confirmed the absence of pathogens. Then, sanitized feathers were ground in a universal cutting mill Pulverisette 19 (Fritsch, Markt Einersheim, Germany) at a rotor rotational speed of 2800 rpm and at a sieve insert size of 1 mm. Ground feathers showed a wide size distribution ranging from 100 μm to a few mm.

A series of rigid polyurethane foams with variation in chicken feather composition were synthesized with a 100% solids castor oil based Polycin M-365 polyol, donated by Vertellus (Greenboro, NC, USA). Amine catalyst Tegoamin^®^ B75, catalyst Kosmos^®^ 29 and surfactant Tegostab^®^ B 8404 were kindly provided from Evonik (Essen, Germany) and distilled water as blowing agent were added to the polyol in part A of the formulation. The polymeric isocyanate obtained from methylene diphenyl isocyanate (PMDI) used in the B-side of the formulation is a commercialized Ongronat^®^ 2100, gently supplied by BorsodChem Zrt (Kazincbarcika, Hungary). It is composed of MDI with highly reactive NCO groups at 4,4 positions. It contains aromatic isocyanates, more reactive than aliphatic one. In Table 1 the physical properties of polyol and PMDI are summarized: 

### 2.2. Biofoam Preparation 

A two component polyurethane system was applied to obtain rigid polyurethane foams. For the RPUF synthesis, M-365, Tegostab B 8404, Tegoamin B75, distilled water, Kosmos 29 and different amounts of ground chicken feathers (0, 2.5, 5, 10 and 15 php, corresponding to RPUF0, RPUF2.5, RPUF5, RPUF10, and RPUF15 samples, respectively) were mixed for 1 min under mechanical stirring of 2000 rpm, in a cardboard beaker. RPUF were produced under the reaction of the premixed polyol with a slight excess of PMDI (NCO/OH ratio of 1.05). Part A and part B (see Table 2) were vigorously mixed for 7 s at room temperature and then poured into a 300 mm × 30 mm × 15 mm closed mold. The density of the biofoams was determined accordingly to ISO 845 [28] and the average density value was obtained as 79.5 ± 2.1 kg/m^3^. All composites were cured at ambient conditions for 24 h.

**Figure 1:**
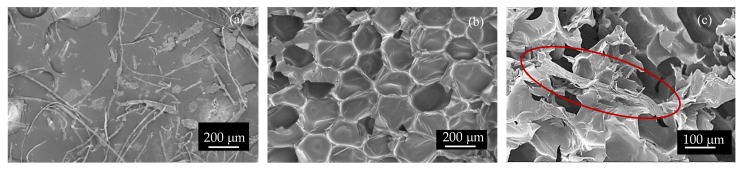
Field-emission scanning electron microscope (FESEM) images of (**a**) ground chicken feathers, (**b**) neat biofoam obtained from castor oil-based polyol and (**c**) a grounded chicken feather (CF) fiber located on several biofoam struts.

In Figure 1, field-emission scanning electron microscope (FESEM) images of (a) ground feathers (b) a neat biofoam and (c) a CF fiber located between foam cells are shown for size comparison purposes. Discussion regarding size distribution will be described in Section 3.2.

### 2.3. Viscosity of CF/Polyol Mixtures

The dynamic viscosity of the polyol with a different amount of ground chicken feathers was determined using a rotational rheometer (AR 2000ex, TA Instruments, New Castle, DE, USA). The measurements were carried out using the plate/plate measuring geometry with a diameter of 40 mm and in the speed range of 1 rpm to 200 s^−1^. The measurements were made at constant temperature of 25 °C.

### 2.4. Characterization of the Biofoams

#### 2.4.1. FESEM

The cell structure of the biofoams was analyzed by scanning electron microscopy of the fractured surface of the composites. The microphotographs were taken with a Carl Zeiss Ultra Plus field-emission scanning electron microscope (FESEM, Oberkochen, Germany) equipped with an energy dispersive X-ray spectrometer (EDXS). Prior to FESEM analysis, samples were Au-coated. Calculations of pore size distributions were examined from a survey of over 30 cells and using ImageJ software from Broken Symmetry software.

#### 2.4.2. Thermal Conductivity

Heat transmission factor for RPU foam samples was determined according to Standard PN-EN ISO 6942:2005 [29]. The tests were carried out under the conditions of exposure of samples to a heat flux with density of 3.0 kW/m^2^. Samples before testing were conditioned at 20 °C and 65% of relative humidity during 24 h.

#### 2.4.3. Compression Test

The compression properties of biofoams in direction perpendicular to the panel surface were performed using a universal testing machine model 3365 (Instron, Norwood, MA, USA) equipped with a 1 KN loading cell and controlled by Bluehill Lite software developed by Instron. The dimension of the foam specimen was 10 mm × 10 mm × 10 mm and the results were calculated using a compression test according to the ISO 844 standard [30]. A crosshead speed of 2 mm/min was used. The compressive strength at 10% and 25% deformation of its original thickness were recorded. The number of tested specimens for the mechanical properties was five for the average calculations. 

#### 2.4.4. Fourier-Transform Infrared (FTIR) Spectroscopy 

FTIR data of the CF, neat RPUF and CF/RPUF was collected using a JASCO FT/IR-4100 (Easton, MD, USA) spectrometer in the range of 4000–400 cm^−1^ and 32 scans being the spectral resolution 1 cm^−1^.

#### 2.4.5. Water Absorption

Water absorption of the biofoams was determined by immersion of the specimens in distilled water at 25 °C for 24 h (ASTM D570-98) [31]. First, rectangular biofoam samples (10 mm × 10 mm × 10 mm) were cut, air-dried at 60 °C for 24 h and cooled in a desiccator and weighed (conditioned weight). Then, samples of the biofoams were soaked in water for 24 h and 48 h and dried and reweighted at fixed time intervals. Three specimens were tested with an analytical balance of 0.1 mg precision and the average and standard deviation were reported. The water absorption rate was calculated using Equation (1):

(1) $$WA\;\left(\%\right)=\;\frac{wet\;weight-conditioned\;weight}{conditioned\;weight\;}\;\times \;100$$

#### 2.4.6. Thermogravimetric Analysis (TGA)

The thermal stability was performed by thermogravimetric analysis using a TGA Q500 (TA Instruments, New Castle, DE, USA). Dynamic measurements were conducted from 25 to 600 °C at a heating rate of 10 °C/min by using constant nitrogen flow of 60 mL/min to prevent thermal oxidation processes of the polymer sample. The temperatures at 5%, 25% and 50% of weight loss were calculated. Sample weight was approximately 10 mg. TGA and DTG (derivative thermogravimetry) curves were plotted with respect to temperature.

## 3. Results and Discussions

### 3.1. Viscosity of the CF/Polyol Premixes.

**Figure 2:**
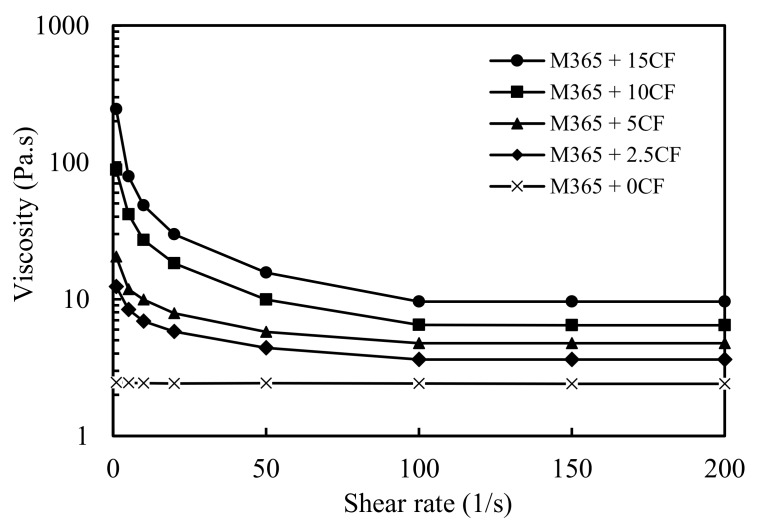
Viscosity of the polyol M-365 and the CF added (php) mixtures.

Viscosity is an important property of raw materials affecting processing and liquid mixing. In foam preparation, low viscosity of the components is a requirement for good mixing during the reaction. The rheological properties of polyol premixes are presented in Figure 2 as the viscosity versus shear rate. The viscosity of the neat polyol showed a typical Newtonian behavior with a constant value of 2.4 Pa·s.

The viscosity of the CF/polyol premixes showed a shear-thinning behavior decreasing the values sharply at low shear rate, and then significantly slowed to reach a relatively stable value. The viscosity of the CF/polyol premixes increased notably with the CF content varying from 2.4 Pa·s for the control sample to 3.6, 4.7, 6.5 and 9.6 Pa·s for 2.5, 5, 10 and 15 php of CF respectively. Among all CF/polyol premixes, that containing 15 php of CF showed a paste-like aspect, possibly hindering a proper mixing of the premix with PMDI.

### 3.2. Morphology of the CF/RPUF Biofoams

One of the most important parameters of RPUF is their cellular structure. Features such as cell size, cell type and cell shape are key attributes in the characterization of PU foams. The effect of incorporation of CF on cellular morphology of the prepared foam samples was studied by electronic microscopy.

**Figure 3:**
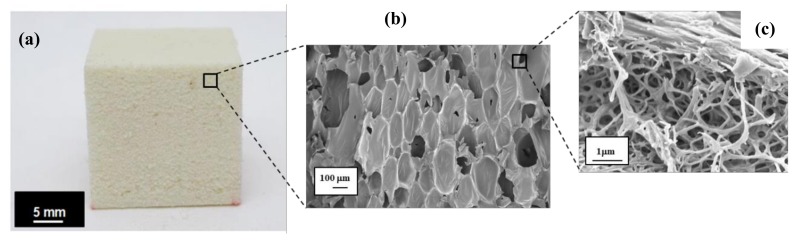
(**a**) Cubic specimen of RPU15 biofoam and FESEM images of the RPUF15 with (**b**) 200× and (**c**) 38,000× magnifications.

Figure 3 depicts an optical image of RPUF15 and FESEM images of RPUF15 at 200× and 38,000× magnifications. FESEM image at 200× magnification revealed the closed-cell porous structure of the RPUF15 and FESEM at a magnification of 38,000× showed the characteristic hollow structure of the ground feathers located on a strut of the foam that enables an improvement on thermal conductivity.

**Figure 4:**
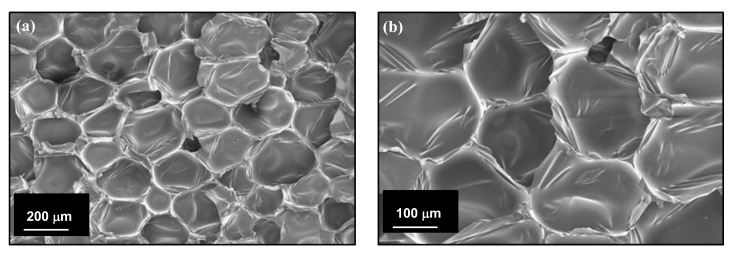

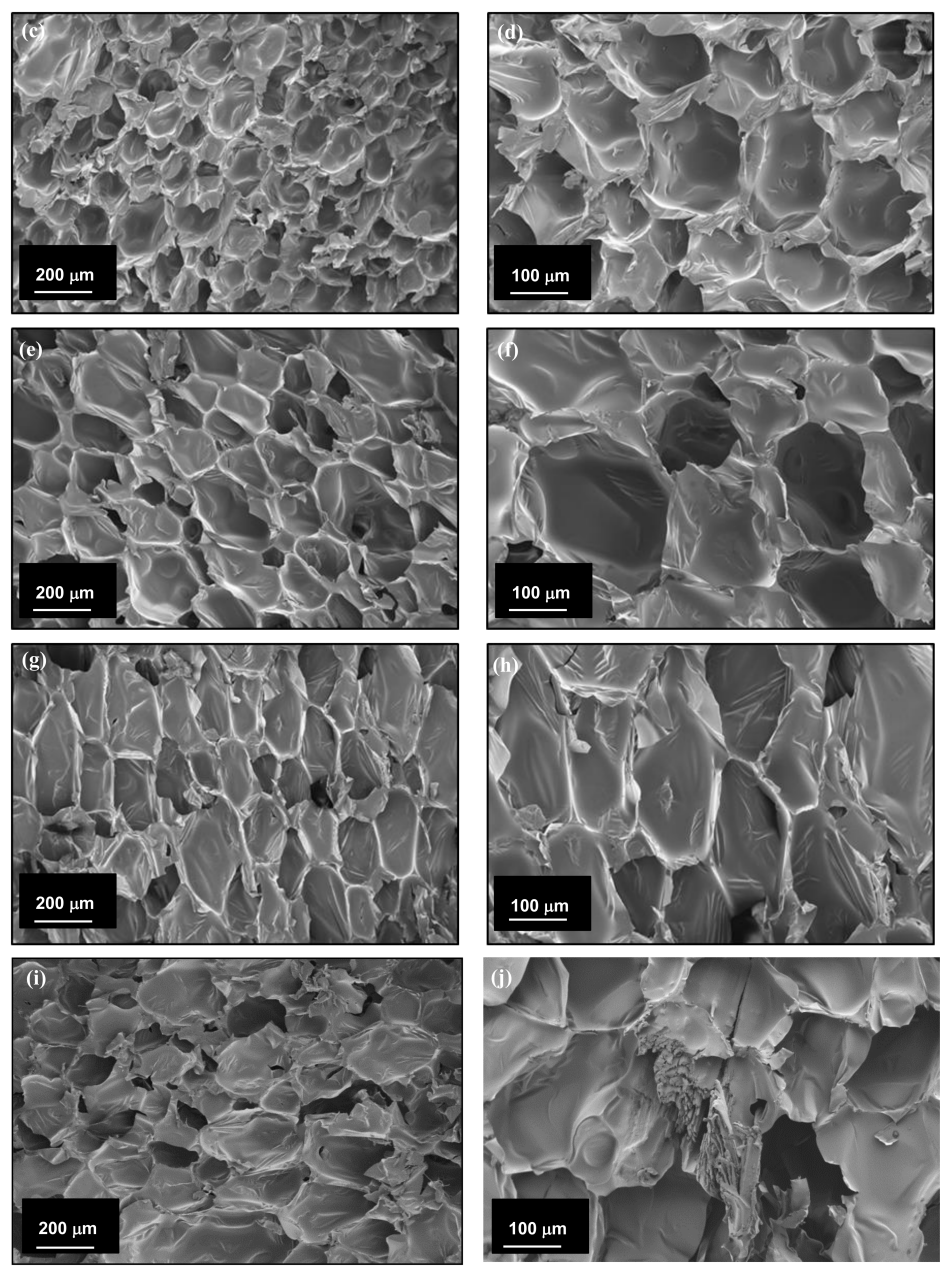
FESEM images of biofoams at 200× (**left**) and 400× magnifications (**right**). (**a**,**b**) RPUF0, (**c**,**d**) RPUF2.5, (**e**,**f**) RPUF5, (**g**,**h**) RPUF10 and (**i**,**j**) RPUF15.

FESEM images of neat RPUF sample and foam samples containing different concentrations of CF are shown in Figure 4 at low and high magnifications. In all biofoams, the principal cell structure was closed with certain broken cells. In RPU0, the cells were predominantly circular with a homogeneous size distribution and diameters ranging from 175 to 265 μm and the addition of CF in RPUF modified the cell wall nucleation towards pentagonal and hexagonal shapes, which distortion increased progressively and widening the cell size distribution of the foams with increasing filler concentration. The shape of the cells varied with CF addition, affecting uniformity of the cellular structure and increasing the number of damaged open cells. According to a previous study, this fact was probably due to the high viscosity of the premixes and the large size of the ground CF that modify notably the growth process of the cells [32]. 

The effect of the CF content in mean cell size of the RPUF was determined from FESEM images using imageJ software and summarized in Table 3. The mean cell size of the neat RPUF0 was 219.22 μm and decreased to 134 and 146 when CF content was 2.5 and 5 php respectively. When the added CF was 10 and 15 php, the mean cell size was 222 and 271 μm, respectively. The decreased cell size in RPUF2.5 and RPUF5 implies that chicken feather fibers act as a nucleating agent. However, the effect is less pronounced at high CF content due to the CF fibers aggregation that are embedded in the struts between cells [33] (see Figure 4j). 

**Figure 5:**
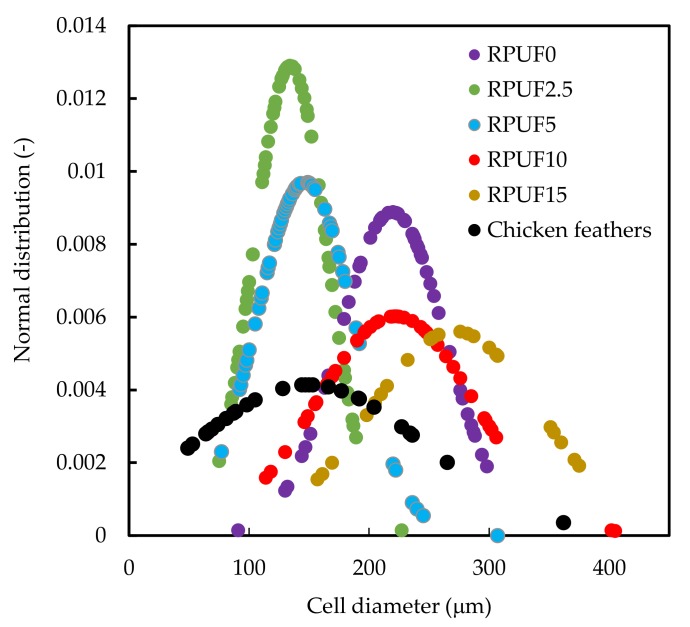
Size distribution of ground chicken feathers and biofoams.

In Figure 5 ground CF size distribution and cell size distributions of biofoams with and without chicken feathers are depicted. The mean size of the CF was 149 mm and showed a broad size distribution ranging from 49 to 362 μm. Regarding the biofoams, the size distribution of the reference, RPUF0, was intermediate among all samples. Biofoams containing 2.5 and 5 php showed narrower distribution and smaller mean size than that of the reference in agreement with similar biofoams [22]. Furthermore, RPUF15 containing a larger amount of filler showed a wider distribution with a larger mean cell size. This effect is mainly due to most of the cells being broken and the cell structure seemed to be partially collapsed.

**Figure 6:**
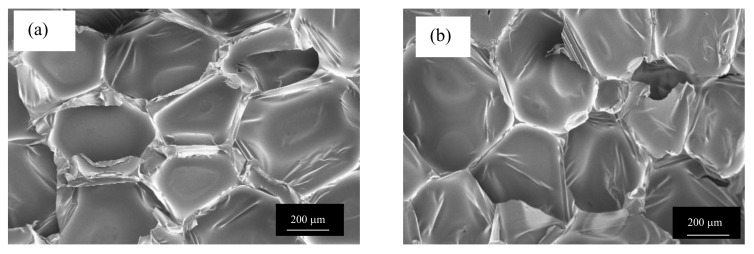
FESEM of biofoams after (**a**) vertical and (**b**) horizontal cell growth.

In the case of PU foams, anisotropy on foam properties caused by the growth directional is an important issue concerning mechanical properties [34]. In this study biofoams were prepared in a closed rectangular mold and foam formation was expected to be similar in all directions. All cells were polyhedral-shaped and were grown in all directions analogously. However, minor morphological differences were observed in perpendicular (vertical and horizontal) growth directions of biofoams in absence of grounded chicken feathers (see Figure 6).

### 3.3. Mechanical Properties of the Biofoams 

The effect of CF on the mechanical properties of the RPUF was investigated measuring the compressive strength and the modulus. As a result of the compression test, compressive modulus and compressive strength at 10% and 25% deformation of pure RPUF foam and CF-filled RPUF were compared and are summarized in Table 4. As it is shown, the compressive modulus and compressive strength at 10% deformation decreased when 2.5 and 5 php of CF were added but slightly recovered when CF were further added up to 10 and 15 php. This was probably due to the high stiffness of the feather fibers. Compressive strength at 25% decreased gradually when CF was increased from 2.5 to 15 php. The interfacial interactions between the feather fibers and the PU foam is a crucial factor affecting the cell structure and the foam performance and it has been demonstrated that the cell structure is an important factor associated with the compressive strength of PU foams [35]. The mechanical properties of RPUF depend on the cell structure, cell thickness and cell distribution in foams and the presence of defects in the cell structure leads to poor mechanical performance [36].

Compressive strength of the biofoams decreased with addition of CF. With increasing CF content, the foam structure became more heterogeneous with unstable and weaker struts. Moreover, low interfacial interaction among feather fibers and the polymer matrix might disturb a stable foam structure and decrease the mechanical properties. S. Czlonka [22] found similar results when 1.5 php CF were added to soybean oil based RPUF.

The decreasing compression strength with CF content is probably due to the heterogeneous dispersion of CF fibers within the polyol first and in the foam then. So, this caused less uniform foam structure as can be seen from FESEM images and decreased mechanical properties. Heterogeneous concentration in some parts of the foams contributed to embrittlement effect of polymer structures and cell walls became weaker. Brittle cell walls could not support the foam structure under high compression loadings and of cell wall rupture was promoted [37].

### 3.4. Thermal Insulation Properties

Thermal insulation is a key parameter in many applications for rigid PU foams. Feathers are inherently insulating due to their structure, which is made by hollow keratin fibers. These hollow cells perform as air and heat insulators. Recently developed needle-punched nonwovens materials reinforced with waste chicken feathers exhibit excellent insulation performance, being thermal conductivity in the range of 0.0313 to 0.04465 W/(m·K) and comparable in values to conventional insulating materials [38].

**Figure 7:**
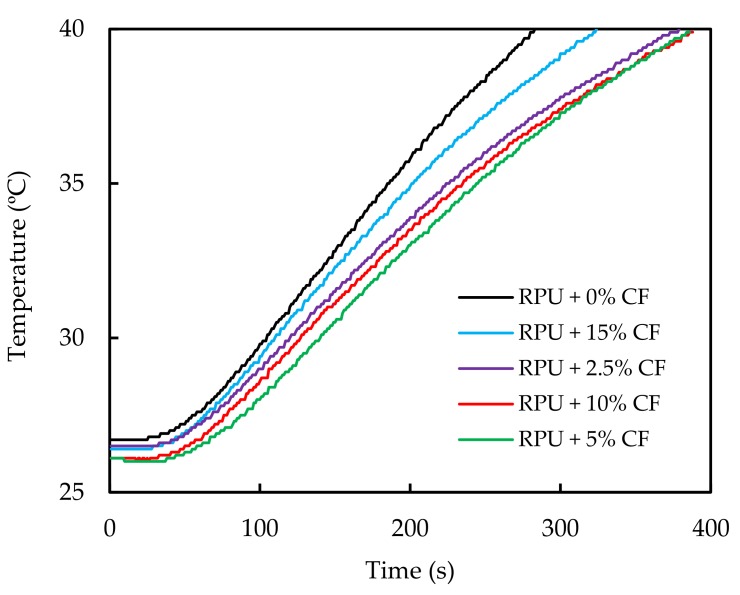
Changes of the temperature of the calorimeter covered with the tested samples under their exposure to a heat flux at an intensity of 3 kW/m^2^.

Thermal conductivity is mainly related with the foam morphology and foam density. Thermal insulation properties of the biofoams are presented in Figure 7 in the form of the temperature dependence of the samples on time of their exposure to the heat flux at intensity of 3 kW/m^2^. When kinetics of heat transfer through RPUF were compared, it could be observed that neat foam with no reinforcing CF showed the fastest temperature variation with time while RPUF containing CF showed an improved thermal insulation property.

Heat transmission factor (HTF) expresses the ability of the tested material to conduct heat and it is defined as the ratio of the density of heat flux that passes through the sample (transmitted heat flux density) to the density of heat flux that falls on the sample (incident heat flux density). Lower HTF value means less ability to conduct heat, i.e., better thermal insulation of the material. Besides, CF exhibit a honeycomb structure [19] in their barbs (see Figure 3), which favors their thermal insulating properties. Therefore the thermal conductivity of CF-reinforced RPUF is expected to decrease with the addition of CF. Heat transmission of biofoams first decreased and then increased (Figure 7). When 2.5, 5 and 10 php CF contents were added, the heat transmission factors were reduced by 20% compared to that of neat biofoam. Due to the addition of CF, the foam cell size became smaller and gave better thermal insulation. Then, as the feather content was increased up to 15 php, many closed cells were broken and air could flow between cells, decreasing the insulation property of the biofoam.

**Figure 8:**
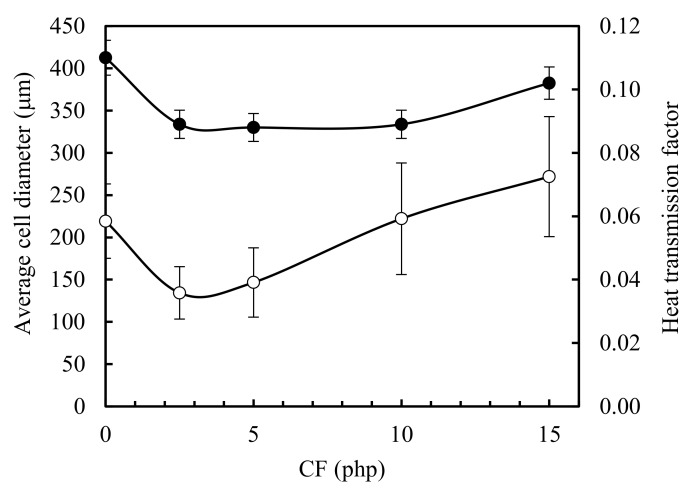
Average cell diameter (white dots) and heat transmission factor (black dots) of the neat foam and the corresponding biofoams as a function of CF content.

Figure 8 depicts the comparison of the heat transmission factor with the average cell diameter of the biofoams as a function of the CF content. The behavior of both curves was similar, suggesting that the foam cells size had a strong effect on the heat transmission of the foams. Foams with smaller average pore size show more struts and walls, which often contribute considerably to the attenuation of thermal radiation [3]. Biofoams with larger pore size show a fractured cell, enabling CO_2_ to flow easily. 

From the HTF values shown in Table 5, it might be concluded that addition of CF into the biofoams leads to a decrease in the heat flux density, improving the thermal insulation properties of the biofoams up to 20%. HTF value of RPUF15 was slightly higher than other CF/RPUF biofoams probably due the low homogeneity of this sample caused by the high viscosity of the part A. 

### 3.5. FTIR Analysis of the Biofoams

**Figure 9:**
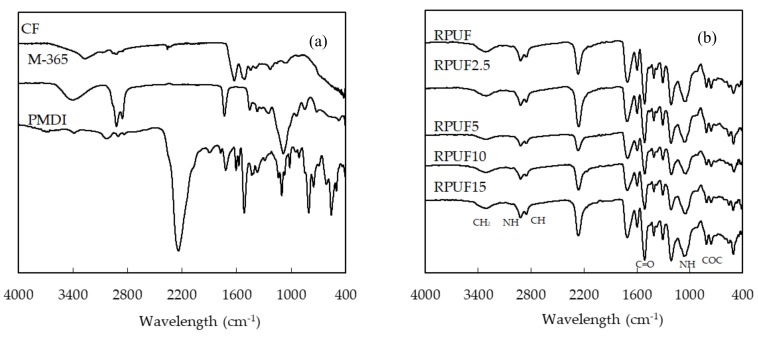
FTIR spectra of (**a**) biofoams and (**b**) CF, M-365 and PMDI.

FTIR spectroscopy was used to analyze the chemical structures of the neat RPUF, CF/RPUF and their main components, CF, polyol and PMDI. In Figure 9a three curves are shown: In the case of CF, the peak at 3210 cm^−1^ was assigned to be the hydrogen bonded N–H (amine) stretching vibrations group with amide C=O groups in the native secondary structure of the keratin [39]. The peak at 2920 cm^−1^ shows the C–H stretch. The peak at 1645 cm^−1^ shows the C=C stretching and the peak at 1516 cm^−1^ shows the C=C bending. On the other hand, the peak at 1229 cm^−1^ is related to C–O (carboxylic acid) originating mainly from different amino acids. The FTIR curve of M-365 displays typical absorption bands of the OH groups at 3380 cm^−1^, ester carbonyl group band from triglyceride of the polyol at 1726 cm^−1^, C–O vibration peak at 1052 cm^−1^ and two peaks at 2912 and 2844 cm^−1^ due to stretching of alkane C–H. In the case of PMDI, at 2240 cm^−1^ the most specific band for isocyanate is observed, representing asymmetrical stretching vibration of isocyanate groups.

FTIR spectra in Figure 9b show the formation of urethane linkage, NH–COO, in the synthesized polyurethanes. The structure of the polyurethanes was confirmed by the presence of three main absorption bands due to the urethane bands formed between castor oil and PMDI and in agreement with previous studies related with foam synthesis from castor oil and 4,4´-methylene diphenyl isocyanate (MDI): 3312 cm^−1^, 1713cm^−1^ and 1509 cm^−1^, corresponding to vibration of N–H (O–H free and amine stretching from urethane group), C=O (stretching vibrations from urethane groups) and N–H (amide II groups) respectively [40]. However, FTIR spectra of all biofoams show many similarities, except for only differences in the intensity of some bands.

The curves corresponding to 2.5, 5, 10 and 15 wt % of CF, show typical bands of the RPUF and CF, difficult to distinguish whether the signal is coming from the polyurethane or from the CF. The presence of the broad band at 3200–3400 cm^−1^ is due to the N–H band of the keratin present in the CF and urethane groups of the polyurethane [41].

### 3.6. Water Absorption

**Figure 10:**
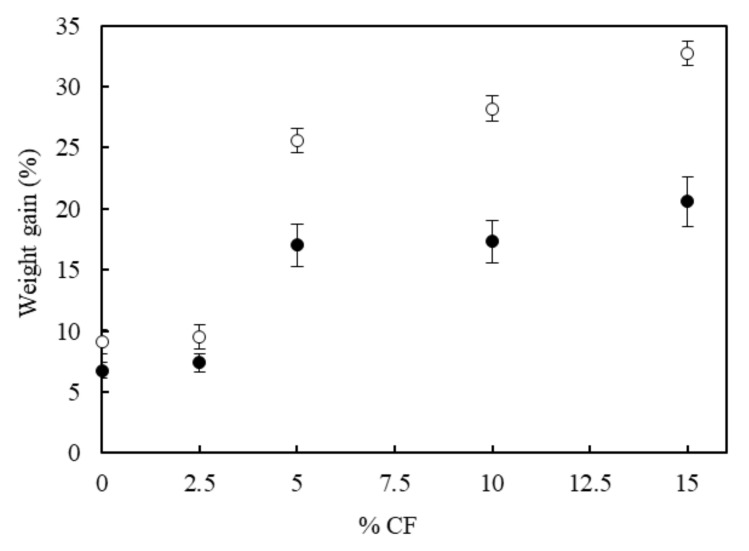
Water absorption of RPUF with different CF content after 24 h (**black circles**) and 48 h (**white circles**) of immersion in water.

Since RPUF are used in the building sector, one of the most important properties to be analyzed in these materials is the water uptake. The amount of water absorption was calculated by the weight difference between the dry sample before the immersion and the sample immersed in water after using Equation (1). Weight gained percentages of the biofoams after immersion in water of 24 and 48 h are shown in Figure 10.

Two main effects are observed: biofoams containing higher CF content absorbed more water and water uptake was more pronounced after immersion of a longer period. The increase of water absorption of different types of CF containing biocomposites has been reported elsewhere [42] and it is related with the presence of hydrophilic groups (45% of total) such as serine and cysteine in the molecular structure of the feathers. The incorporation of CF, partially hydrophilic, increased greatly the water content of the biofoams. The neat biofoam absorbed 9% after 48 h and increased up to 32.7% when the CF content was 15 php. These values are much lower than 50% of water uptaken by foams reinforced with natural fibers such as hemp [43] and confirmed the hydrophilic nature of the CF that will be observed during the initial stages of the thermogravimetric degradation analysis in Section 3.7.

### 3.7. Thermogravimetric Analysis 

**Figure 11:**
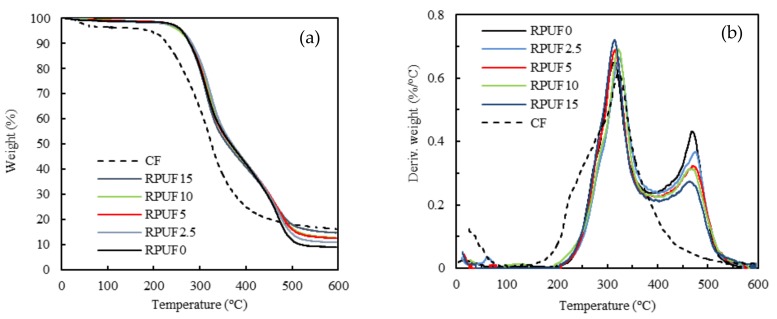
(**a**) TGA and (**b**) DTG of RPUF control and CF/RPUF with 2.5%–15% CF php.

The thermogravimetric analysis (TGA-DTG) under nitrogen atmosphere of neat RPUF and CF/RPUF samples is shown in Figure 11a,b. The weight loss of the CF is represented in both figures as reference. In the case of the thermal degradation of CF, see Figure 11a, three weight-loss steps are observed. In the first stage, from 25 to 250 °C, the weight loss is due to the evaporation of water molecules absorbed by the hydrophilic moieties of the CF present in polar amino acids such as serine. The second weight loss, between 250 and 400 °C, shows a higher rate and is related with the skeletal and chemical degradation associated with the destruction of disulfide bonds and the elimination of H_2_S originating from amino acid cysteine [44]. The third weight loss occurred from 400 °C onwards and was associated with the decomposition of the keratin. For pure CF, 16.9 wt % of carbonized residue was left, similar to previous reports [45]. In the case of the neat biofoams, an insignificant mass loss of 1.5% due to the moisture loss and evaporation of some small molecules from 25 to 200 °C was observed. The urethane group started decomposing around 200 °C and biofoams showed a two-stage decay related with two maximums (see, DTG curves in Figure 11b); the first at 320 °C, similar to the maximum degradation rate of CF and a second maximum at 465 °C.

Addition of 2.5 and 5 php CF hardly improved the thermal stability of the RPUF0, starting to degrade at similar temperatures. On the contrary, addition of higher CF content caused a thermal degradation at earlier temperatures, related with low thermal stability of the CF. The shape of the mass loss curves of the CF/RPUF biofoams is similar with an increase of CF content. At higher temperatures the recorded weight loss is related to the polyol backbone degradation [46].

Between 500 and 600 °C, biofoams containing different CF loadings showed intermediate mass loss between the mass loss of CF and that of control biofoam. In this range of temperatures, CF showed the highest char level (see Table 6) even started to degrade at a lower temperature than biofoams.

DTG of CF and neat RPUF (Figure 11b) showed maximum weight losses at 323 and 313 °C respectively and intermediate values for biofoams. The DTG curve of CF showed a single wide peak in the range of 200–350 °C whereas biofoams show two peaks related to the dissociations of urethane bonds (310–330 °C) and decomposition of polyol segments (465–475 °C).

In Table 6, the 5%, 25% and 50% weight-loss temperatures and residual mass after 600 °C are listed for RPUF0, RPUF2.5, RPUF5, RPUF10, RPUF15 and CF. It was observed that with the addition of small amounts of CF, the onset of the thermal degradation maintained unchanged. However, when further CF was added to the RPUF, the thermal stability behavior varied. This can be attributed to the fact that in CF/RPUF the protein content was significant and the thermal degradation of the foams was more related to the keratin degradation [47].

## 4. Conclusions

In this work, polyurethane foams made from bio-based polyols and reinforced with chicken feathers were successfully developed and their morphology, thermal conductivity, thermal stability and mechanical properties were investigated. The cellular structure of these biofoams presented low anisotropy in polygonal shaped cells. Mechanically, the compression strength was decreased with the addition of CF. The thermogravimetric curves revealed that the thermal stability of the biofoams containing chicken feathers showed a minor effect comparing to that of neat biofoams. Despite the mechanical and thermal properties were penalized by the incorporation of CF, the resulting biofoams showed an improved thermal insulation up to 20%. 

The manufacturing of biofoams containing castor oil based polyols and chicken feathers with up to 45% of bio-based components and improved thermal insulating properties would contribute to a more efficient use of natural resources and take advantage of the later raw material that is produced in huge amounts worldwide and is currently underutilized by the poultry industry. It is intended that the use of materials from renewable resources contribute to sustainability and a reduction in the environmental impact associated with the incineration or disposing of poultry feathers into landfills.

## Figures and Tables

**Table 1:** Properties of the polyol and polymeric isocyanate obtained from methylene diphenyl isocyanate (PMDI).

Compound	Property	Value
Polyol	Density (25 °C)	1.02 g/cm^3^
	Viscosity (25 °C)	2.0 Pa·s
	Acid Value	1
	Equivalent weight	154
	Functionality	4
	Hydroxyl value	365
PMDI	Density (25 °C)	1.23 g/cm^3^
	Viscosity (25 °C)	0.17–0.23 Pa·s
	NCO content	30–32
	Functionality	2.6–2.7

**Table 2:** Formulations of bio-based RPUF. All formulations are based on 100 parts per weight of polyol (php).

	Component	RPUF0	RPUF2.5	RPUF5	RPUF10	RPUF15
Part A	Polyol M-365	100	100	100	100	100
	Surfactant (Tegostab B 8404)	3	3	3	3	3
	Amine (Tegoamin B75)	0.5	0.5	0.5	0.5	0.5
	Blowing agent (distilled water)	3	3	3	3	3
	Catalyst (Kosmos 29)	0.4	0.4	0.4	0.4	0.4
	Chicken feathers	0	2.5	5	10	15
Part B	PMDI (Ongronat^®^ 2100)	135	135	135	135	135

**Table 3:** Mean cell size of rigid polyurethane foam (RPUF).

Sample	Cell Size (μm)
RPUF0	219 ± 44
RPUF2.5	134 ± 31
RPUF5	146 ± 41
RPUF10	222 ± 66
RPUF15	271 ± 71

**Table 4:** Mechanical properties of the biofoams.

Sample	Young’s Modulus (MPa)	Compressive Strength (10% def; KPa)	Compressive Strength (25% def; KPa)
RPUF0	3.2 ± 0.4	230.2 ± 13.5	348.2 ± 11.6
RPUF2.5	2.2 ± 0.2	177.1 ± 3.7	271.1 ± 7.6
RPUF5	1.7 ± 0.5	129.8 ± 26.6	234.5 ± 27.1
RPUF10	1.8 ± 0.4	131.1 ± 34.2	229.5 ± 40.6
RPUF15	2.2 ± 0.3	150.5 ± 13.4	215.5 ± 21.4

**Table 5:** Heat transmission factor of RPUF and CF /RPUF biofoams.

Tested RPUF	Heat Transmission Factors (HTF)
RPUF0	0.110
RPUF2.5	0.089
RPUF5	0.088
RPUF10	0.089
RPUF15	0.102

**Table 6:** Thermal characterization of CF, RPUF0 and the CF/RPUF biofoams.

Sample	T (5%; °C)	T (25%; °C)	T (50%; °C)	Residual Mass after 600 °C (%)
RPUF0	261	308	367	9.08
RPUF2.5	262	315	370	10.84
RPUF5	262	310	366	12.52
RPUF10	257	313	365	12.58
RPUF15	259	307	359	14.73
CF	196	271	325	16.24
